# Impact of Somatic Development and Course of Osteogenesis Imperfecta on FGF23 Levels in Children

**DOI:** 10.3390/ijms26136007

**Published:** 2025-06-23

**Authors:** Agnieszka Byrwa-Sztaba, Elżbieta Jakubowska-Pietkiewicz

**Affiliations:** Department of Pediatrics, Neonatal Pathology and Metabolic Bone Diseases of Medical University of Lodz, 91-738 Lodz, Poland; elzbieta.jakubowska-pietkiewicz@umed.lodz.pl

**Keywords:** osteogenesis imperfecta, FGF23, somatic development, children

## Abstract

Osteogenesis imperfecta (OI) is a rare bone dysplasia that occurs with a frequency of 1/15,000–20,000 live births. It is characterized by increased susceptibility of bone fractures, skeletal deformities, low stature, and low bone mass. It results in impaired production of type I collagen. About 90% of people with OI have heterozygous mutations in the *COL1A1* and *COL1A2* genes. Fibroblast growth factor 23 (FGF23) is a protein involved in the regulation of phosphate and 1,25-dihydroxyvitamin D_3_ metabolism on a negative feedback basis. FGF23 is secreted by osteocytes in response to increased serum calcitriol and phosphorus. The purpose of this study was to evaluate the concentration of FGF23 among children with osteogenesis imperfecta and the differences in reference values in a healthy population of children and adolescents. Then, this study sought to evaluate how the course of osteogenesis imperfecta, including type of disease, number of bone fractures, and bone mineral density, are related to FGF23 concentration. The study included 47 children aged 3 to 17 years with a diagnosis of osteogenesis imperfecta, confirmed by genetic tests. The patients were hospitalized at the Department from August 2019 to September 2020 and were treated with intravenous infusions of sodium pamidronate. The course of the disease was analyzed, including the number of bone fractures, clinical symptoms, and anthropometric parameters, and bone densitometry was performed by dual X-ray absorptiometry (DXA) in Total Body Less Head (TBLH) and Spine options with Z-score evaluation. FGF23 concentration was determined by the ELISA method. The study was prospective in nature. Results: The mean level of FGF23 in the study group of patients was 645.09 pg/mL and was within the reference values for the developmental age population. There was no significant correlation between FGF23 concentration and anthropometric measurements: body weight (*p* = 0.267), height (*p* = 0.429), gender (*p* = 0.291), or pubertal stage (*p* = 0.223) in the study group of patients. FGF23 levels were not related to the number of fractures (*p* = 0.749), the number of sodium pamidronate cycles administered (*p* = 0.580), bone mineral density parameters (Z-score), the form of osteogenesis imperfecta (*p* = 0.156), or the genetic test result (*p* = 0.573). FGF23 levels decrease with age (r = −0.32, *p* = 0.030) and BMI (r = −0.34, *p* = 0.020). The level of FGF23 in patients with osteogenesis imperfecta is lower among older children and those having a higher BMI. This index cannot be a diagnostic tool in this group of patients, for no differences were found between the concentrations in patients with osteogenesis imperfecta and the developmental age population.

## 1. Introduction

Osteogenesis imperfecta (OI) is a heterogeneous group of rare diseases (OMIM 166200, 259420, 166220, 610967, 613982) classified as bone dysplasia, occurring with a frequency of 1/15,000–20,000 live births. It is characterized by increased susceptibility of bones fractures, skeletal deformities, low stature, and low bone mass. Due to impaired production of type I collagen in various tissues, patients with OI may also present other clinical manifestations, such as dentinogenesis imperfecta, blue sclerae, hearing loss, reduced respiratory capacity, and valvular regurgitation in the heart [1]. In recent decades, tremendous progress has been made in identifying the genes and molecular mechanisms underlying OI. The classic clinical classification of Sillence is still relevant but a functional classification based on metabolic mechanisms has been proposed, with the following groupings: group A—defects in collagen synthesis, structure or processing, group B—defects in collagen modification, group C—defects in collagen folding and cross-linking, group D—impaired bone mineralization, and group E—defects in collagen-deficient osteoblast development [2]. Approximately 90% of people with OI have heterozygous mutations in the *COL1A1* and *COL1A2* genes, with a dominant pattern of inheritance or sporadic mutations [3]. The diagnosis of osteogenesis imperfecta is currently made on the basis of clinical data and can be confirmed by genetic testing. Imaging diagnosis in the form of conventional radiography remains the most common imaging modality in osteogenesis imperfecta. The main radiographic features include generalized osteopenia/osteoporosis, bone deformities, and fractures. Bone densitometry is currently the most available technique for assessing bone mineral density, which is reduced in all forms [4].

Treatment of patients with OI is based on the nature and severity of symptoms, and the goal of therapy is to prevent fractures and disability, as well as to improve function and quality of life [5]. It should be noted that the goals of OI treatment go beyond fracture risk reduction and include maximizing growth and mobility and treating extra-skeletal complications [6]. While intravenous bisphosphonates remain the mainstay of OI treatment, new therapeutic strategies such as sclerostin inhibitory antibodies and TGF-β growth factor are being explored to address not only low bone mineral density, but also to reduce the incidence and number of bone fractures [7]. In turn, drug treatment complements physiotherapy, rehabilitation, and orthopedic care [8]. Pamidronate therapy in children and adolescents with OI leads to a significant increase in bone mineral mass, which is due to both an increase in bone size and bone mineral density [9].

The fibroblast growth factor-23 (FGF23) discovery in 2000 and its role became a breakthrough in the regulation of bone and mineral homeostasis. FGF23 is a protein encoded by the FGF23 gene and is a humoral factor, of which 22 have been described in humans. The strong link between FGF23 and vitamin D metabolism became apparent soon after the initial description of FGF23 as a putative phosphaturic hormone. It turned out that this factor was responsible for the genetic form of autosomal-dominant hypophosphatemic rickets (ADHRs) and a hereditary kidney disease causing phosphate loss [10,11,12]. The regulatory mechanisms of FGF23 synthesis have not been fully elucidated. It has been postulated that FGF23 regulates phosphate and vitamin D metabolism by binding to the FGFR–Klotho complex. In proximal renal tubules, FGF23 inhibits membrane expression of sodium–phosphate cotransporters, which mediate the reabsorption of filtered phosphate in urine. In addition, FGF23 inhibits the expression of 1α-hydroxylase in proximal tubules, a key enzyme responsible for vitamin D hormone production. Fibroblast growth factor decreases phosphate and 1,25(OH)_2_D, while phosphate and 1,25(OH)_2_D increase its production [13].

FGF23 production is regulated by various local and systemic factors. Systemic regulators include, in addition to phosphate and 1,25(OH)_2_D, the following: parathormone, insulin, iron, and inflammation. Whether they play a physiological role in regulating its levels is not clarified [14]. Local negative regulators of FGF23 production include a phosphate-regulating gene homologous to endopeptidase on X chromosome (*PHEX*) or dentin matrix protein 1 (DMP1). Their inactivation causes overproduction of FGF23 and hypophosphatemia. It has also been suggested that sclerostin has a role in regulating FGF23 production, which may link two osteocyte functions: control of bone mass and phosphate homeostasis [15].

There are also reports that FGF23 is a pleiotropic hormone, as it additionally exhibits effects on receptors located in the heart and may contribute to cardiovascular mortality and left ventricular hypertrophy in chronic kidney disease [16]. In the pediatric population, FGF23 may affect bone growth, as well as cause high and age-dependent serum phosphate levels that may affect bone turnover. However, the interaction between FGF23 and vitamin D in children is largely unknown and the interpretation of the results poses many difficulties [17]. 

### Aim of the Study

The aim of this study was to evaluate FGF23 levels among children with osteogenesis imperfecta and their differences from reference values in a healthy population of children and adolescents. Then, this study sought to evaluate how the course of the disease, including its type, genetic mutations, number of bone fractures, and bone mineral density, are related to FGF23 concentration.

## 2. Results

Statistical analysis included data collected from 47 patients. The mean age of the patients was M = 8.83 years (SD = 3.76 years). Table 1 summarizes the most common clinical manifestations of congenital bone fracture in the studied group of children. It shows that 66% of the children in the study group had vertebral fractures, and more than half (53.2%) had low stature. Meanwhile, 28 patients underwent orthopedic procedures to correct bone axes.

The mean body weight was 25.18 kg, with a median of 18 kg and a range of values between 9.70 kg and 73 kg. The distribution of the data for body weight was characterized by an apparent right-handedness, meaning that most measurements were below the mean. The mean height/length of the patients studied was 115.09 cm with a range of Comvalues between 53 cm and 175 cm. The mean BMI was 17.90, with values ranging from 11.89 to 37.35. The body weight of the studied patients was plotted on centile grids. Nearly half of the children studied (48.9%) had a body weight below the 3rd percentile. Slightly more than half of the patients studied had body height/length below the 3rd percentile (53.2% of the group). BMI values were plotted on centile grids. More than half of the study group (61.7%) had a normal BMI. The concise data on body weight, height/length, and BMI is shown in Table 2.

In the next step, the pubertal stage of the studied group of children was analyzed according to the Tanner scale. The vast majority of patients were in pubertal phase I (70.2% of the group), phase II affected 10.6% of the group, and phase III affected 8.6% of the group.

More than half of the study group (65.9%) had a mutation in the *COL1A1* gene upon genetic testing, while a mutation in the *COL1A2* gene affected one in four patients (24.4% of the group). Three patients (7.3%) had a confirmed mutation in the *IFITM5* gene, while one patient had a mutation in *SERPINF1* as presented in Table 3.

The most common type of OI in the study group was type I (51.1% of the group), followed by type III (34.0% of the group).

Parameters such as the number of cycles, number of fractures, and bone mineral density (Z-score) were also analyzed. In the study group, the average number of cycles was M = 12.04 (with a range of 1 to 37 cycles). The number of fractures averaged at M = 9.46, with a median of Me = 6.50 and a range of 1 to 50 fractures. A strong right-skewness of the distribution of the number of fractures is evident, meaning that most of the results were below the mean.

The values of Z-score Spine and Z-score Total Body Less Head parameters were calculated. The mean value of Z-score Spine was M = −2.53, while Z-score Total Body was M = −2.24. Individual test results confirmed osteoporosis in 25 cases (53.2% of the study group).

**Figure 1:**
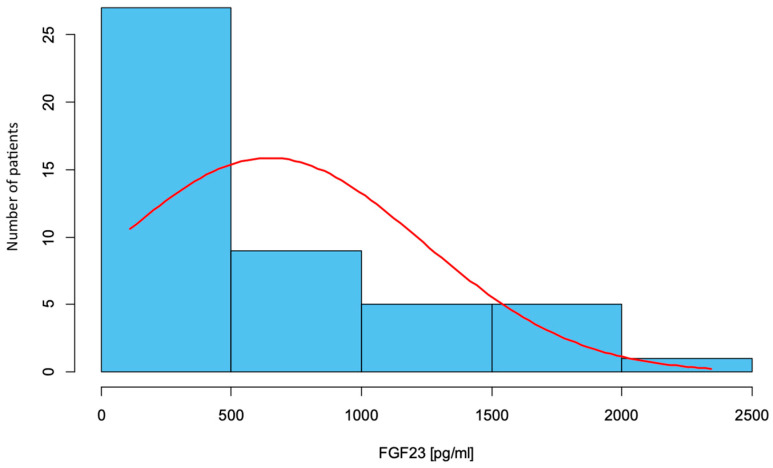
FGF23 concentration in the study group of children with osteogenesis imperfecta.

The study analyzed the results of FGF23 levels in a group of children and adolescents with osteogenesis imperfecta. The mean FGF23 concentration was M = 645.09 pg/mL, while the median took the value Me = 374.00 pg/mL. A strong right-handedness of the data distribution for FGF23 is evident, meaning that most of the results were below the mean. The lowest result obtained was 113 pg/mL, while the highest was 2342.00 pg/mL. Elevated FGF23 levels were observed in 6 patients in the study group of children with OI. The results of FGF23 levels are shown as a histogram in Figure 1.

**Figure 2:**
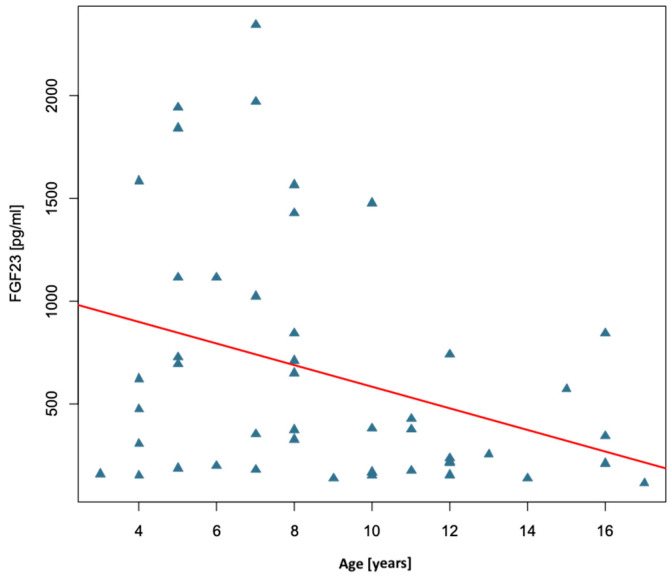
Relationship between patient age and FGF23 concentration in the study group of children with osteogenesis imperfecta.

**Figure 3:**
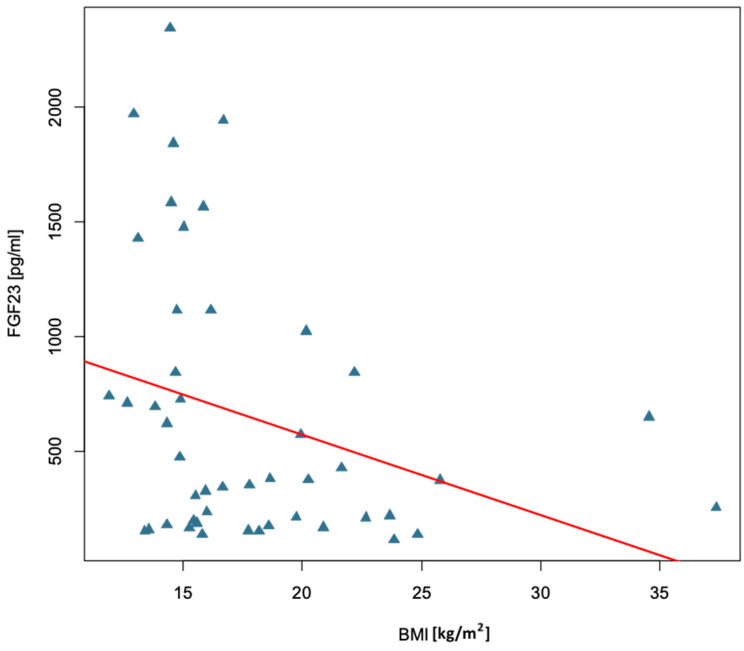
Relationship between BMI and FGF23 concentration in the study group of children with osteogenesis imperfecta.

The next part of the analysis compares the concentration of FGF23 in relation to individual anthropometric, biochemical, and densitometric parameters of the studied group of children with congenital bone fragility. It was checked whether FGF23 concentration depends on the gender of the studied children. The median FGF23 concentration among girls was Me = 473.00 pg/mL, while among boys it was 278.00 pg/mL, but there was no statistically significant difference in FGF23 concentration between girls and boys (*p* = 0.291). Next, we checked whether there was a correlation between FGF23 concentration and age, anthropometric parameters, and bone mineral density parameters in the study group. A statistically significant correlation of FGF23 concentration with age (r = −0.32, *p* = 0.030) and BMI (r = −0.34, *p* = 0.020) was confirmed. In both cases, the correlation coefficient had moderate strength and took a negative value, which means that FGF23 concentration decreased with age and increasing BMI, as shown in Figure 2 and Figure 3. No statistically significant association was confirmed between FGF23 concentration and individual parameters of bone mineral density, body weight, or height/length, as shown in Table 4.

**Figure 4:**
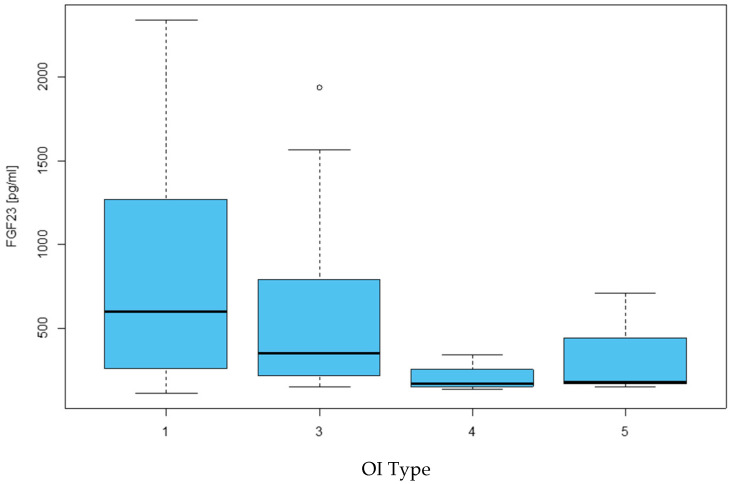
Relationship between FGF23 concentration and the type of osteogenesis imperfecta in the study group of children with osteogenesis imperfecta.

We checked whether FGF23 levels were associated with the type of OI of the children studied. The median concentration of FGF23 in the group of patients with type I was Me = 596.00 pg/mL, Me = 349.00 pg/mL for type III, while for children with types IV and V, it was Me = 167.00 pg/mL and Me = 179.00 pg/mL, respectively. But there was no statistically significant difference in the concentration of FGF23 and the type of OI of the studied patients (*p* = 0.156), as presented in Table 5 and Figure 4.

It was also checked whether FGF23 concentration depends on the pubertal stage of the studied children. The median FGF23 concentration in the group of patients who were in stage I was Me = 473.00 pg/mL, Me = 425.00 pg/mL for stage II, and for children in stages III and V, it was Me = 280.00 pg/mL and Me = 209.00 pg/mL, respectively. But there was no statistically significant difference between the FGF23 concentration and the pubertal stage of the studied patients (*p* = 0.223).

## 3. Discussion

Osteogenesis imperfecta belongs to bone dysplasia and is a genetic disorder of connective tissue caused by abnormalities in the synthesis or processing of type I collagen. Clinical manifestations range from mild with a nearly asymptomatic form to severe with progressive bone deformities. Forty-seven patients of both sexes aged 3 to 17 years with a diagnosis of congenital bone fracture type I, III, IV, V, and VI were enrolled in the study group. One complete course of treatment was analyzed for each patient in the study group. The number of cycles varies widely. Due to the different stages of treatment, the study included patients who were just starting therapy and those who were already receiving maintenance treatment. Analysis began by presenting the most common clinical symptoms accompanying congenital bone fracture in the study group of children. Anthropometric measurements in the study group were plotted on centile grids developed by the Maternal and Child Health Center Institute [18]. It was shown that the majority of patients with OI were characterized by short stature.

Short stature is one of the primary symptoms of congenital bone fracture, with a decrease in the height of vertebral bodies occurring most often in the thoracic, then in the lumbar [19,20]. The results of our own study confirm that patients with OI are low in height, as most of them are below the 3rd percentile for age. In the study group, 53.2% of patients present this clinical sign, which is consistent with the literature of Castelein et al. and Zeitlin et al. [20,21]. Osteogenesis imperfecta is associated with short stature, the reduction of which is mild, severe, and moderate in types I, III, and IV of OI, respectively [22]. The body height of patients with osteogenesis imperfecta type I is considered normal or only slightly reduced, but studies show that children with osteogenesis imperfecta type I are smaller from the start than their healthy peers [23]. A reduction in body height to the greatest extent and with the greatest variability is observed in osteogenesis imperfecta type III/IV and in patients with qualitative collagen defects [24]. Body proportion abnormalities were observed in all patients with type III OI in the present study.

Vertebral fractures in the study group occur in 31 (66% of the study group) children with OI and most often involve the thoracic segment of the spine. Deformities of the skeletal system in the form of, among others, a puckered chest, arched upper and lower limbs as well as lower extremities, prominent frontal cusps, a triangular facial appearance, shortened limb lengths, or scoliosis affect as many as 32 patients (68% of those studied), as also emphasized by other authors [8,25].

The hallmark of osteogenesis imperfecta is an increased susceptibility to bone fractures. The study showed that all had fractures of long bones in both the lower and upper extremities, and the number of fractures itself varies widely. Bone fractures usually involve the epiphyses of long bones, and it is common for a fracture to occur several times in the same place, which usually results in orthopedic treatment [26]. In the study group, osteoporosis, according to the criteria recommended by the International Society for Clinical Densitometry [27], was diagnosed in more than half of the patients (53.2%). It should be noted that some of the patients had Z-scores within the reference value range of −1 to +1 in both Total Body Less Head and Spine projections, and the majority of this group was diagnosed with Type I OI or was on maintenance treatment. In contrast, the remaining 16 patients were diagnosed with reduced bone mineral density (formerly osteopenia).

In addition, this group is characterized by weight deficiency, with a median BMI of 15.95 indicating weight deficiency. A retrospective analysis of the medical records of patients with osteogenesis imperfecta types I and III was conducted at our clinic, which found an abnormal BMI in 41.67%, of whom 37.78% were underweight, 48.89% were overweight, and 13.33% were obese [28]. This difference is likely due to the number of patients with a specific type of osteogenesis imperfecta; in our study, patients with type I predominated (51.5%). Obesity is a problem in the majority of patients with OI, with patients with type III being the most affected [29]. According to the Polish Society for the Treatment of Obesity, in Poland, overweight or obesity is present in 12.2% of pre-school boys and 10% of pre-school girls, as well as in 18.5% of school-aged boys and 14.3% of school-aged girls [30]. This means that obesity and overweight in children with OI are more common than in the healthy population.

In the study group, more than half of the patients (59.6%) had undergone corrective surgery, mainly to the long bones of the upper and lower extremities. Patients with osteogenesis imperfecta are recommended to receive fracture treatment with rapid mobilization and placement of intramedullary rods to prevent or correct long bone deformity [31]. In the study group, more than half of the patients were diagnosed with type I of OI, which, according to Silence’s classification, is a mild type, followed by type III being severe, while types IV, V, and VI are of moderate severity. The vast majority of cases were confirmed by genetic testing, with a predominant mutation of the *COL1A1* gene, which is consistent with the literature [3,32,33]. Our study shows that FGF23 levels correlate negatively with age. This means that the older the child is, the FGF23 concentration decreases, which is in line with the observations of Stańczyk et al. [34]. It was also shown that there is a negative correlation between FGF23 and BMI, meaning that the higher the BMI, the lower the FGF23. Obesity may increase FGF23 production in the absence of chronic kidney disease. In the present study, the percentage of overweight and obese children was 19.2%, higher than in the healthy population. One study evaluating normotensive African-American adolescents examined whether FGF23 concentrations were higher in obese compared to normal-weight adolescents. It was shown that FGF23 concentrations were higher in obese compared to normal-weight subjects [35]. Perhaps the effect on the relationship between BMI and FGF23 concentration in the study group depends on the number of overweight and obese children. The value of the factor is not related to gene mutation or type of osteogenesis imperfecta. It should be noted that the size of the subgroups works against the results. The subgroup with a known mutation in the IFITM5 gene has only 3 patients. There was no statistically significant difference between FGF23 levels and the pubertal stage of the patients studied. In contrast, one study tested the association of FGF23 and pubertal period in patients with hypophosphatemia and in healthy children. Concentrations of iFGF23 (full-length) were higher in healthy infants than in prepubertal (*p* < 0.01) and post-pubertal (*p* < 0.05) children, and pubertal subjects showed higher values (*p* < 0.05) than post-pubertal subjects. Serum iFGF23 and phosphate concentrations did not differ by sex, age of menarche, and time after menarche. Concentrations of iFGF23 were higher (*p* < 0.0001) in patients with X-chromosome-associated hypophosphatemia than in healthy subjects according to chronological age and pubertal development. In all patients, iFGF23 levels were above 40 pg/mL [36]. No such relationship was observed in the OI patients studied. The study did not show a relationship between FGF23 levels and bone mineral density parameters (Z-score) in a group of children with osteogenesis imperfecta. One study evaluated the effect of intravenous pamidronate administration on serum FGF23 levels in patients with osteogenesis imperfecta treated with two cycles of 3-day pamidronate infusion. It was observed that FGF23 levels decreased significantly [37]. Our study included analysis of only one cycle of pamidronate sodium treatment. FGF23 concentration was assessed only before administration of the drug, which constitutes a certain limitation in comparing the results of these two studies. However, it cannot be excluded that inhibition of osteoclast function may play a role in suppressing FGF23 levels.

## 4. Material and Methods

The prospective research project was conducted in the Department of Pediatrics, Neonatal Pathology and Metabolic Bone Diseases at the Medical University of Lodz from August 2019 to September 2020. The study was approved by the Bioethics Committee No. RNN/37/19/KE dated 15 January 2019. The study group included 47 patients of both sexes: 25 girls and 22 boys in the age range of 3–17 years with a diagnosis of congenital bone fracture type I, III, IV, V, and VI who were treated with intravenous infusions of bisphosphonates and hospitalized at least once from August 2019 to September 2020. During this time, each patient had a blood sample taken for the determination of fibroblast growth factor FGF23. A blood sample was taken prior to intravenous bisphosphonate administration in order to assess FGF23 concentration. One full course of treatment was analyzed in each patient.

Based on the available medical records of the subjects in the study group, the following parameters were analyzed: gender, age, number of fractures, number of cycles of the drug administered, and anthropometric measurements: body height in cm was measured with a stadiometer for standing patients while body length in cm was measured for lying patients; body weight in kg was measured using a doctor’s scale while for patients in a wheelchair, a chair scale was used. Measurements of weight, height/body length, and BMI were plotted on centile grids developed by the Institute of Mother and Child Health Center [18]. Pubertal stage was measured with the Tanner scale [38], fibroblast growth factor FGF23 concentration was measured with the ELISA immunohistochemical method using streptavidin–horseradish peroxidase (HRP) reagent, ELISA Kit, SunRed Biotechnology Company, catalog no. 201-12-0060, 2019, Shanghai, China. The result of bone densitometry in Total Body Less Head and Spine projections (performed on an outpatient basis in the year covering the review of medical records) was obtained via dual-energy X-ray absorptiometry (DXA) technique on a Horizon Wi (S/N 200800) [27]. The reference range for the FGF23 concentration test is 10–1500 pg/mL.

Statistical analysis was performed using statistical program R, version 4.2.1. (R Core Team (2022). R: Language and environment for statistical computing by R Foundation for Statistical Computing, Vienna, Austria). A significance level of α = 0.05 was used in the calculations. Normality of the distribution was analyzed using the Shapiro–Wilk test and visual assessment of histograms. Comparison of FGF23 concentrations in relation to selected qualitative variables was based on Mann–Whitney U test and Kruskal–Wallis test, according to the number of subgroups compared. When comparing 2 subgroups, the median difference (MD) was additionally calculated, taking into account the 95% confidence interval (CI). Analysis of the relationship between FGF23 concentration and quantitative variables was based on Spearman’s correlation coefficient. In interpreting the correlation coefficient, values between 0.3 and 0.5 in absolute terms were taken as the average level of correlation, values between 0.5 and 0.7 in absolute terms were considered good correlation, and values higher than 0.7 in absolute terms were considered very strong correlation. Thus, values of the correlation coefficient less than 0.3 in absolute terms were considered poor correlation [39].

## 5. Conclusions

FGF23 concentration in children with OI decreases with age and increase in weight-for-height index. There was no relationship between FGF23 concentration and the type of osteogenesis imperfecta, its clinical course, and bone mineral density in the study group of children and adolescents.

## Figures and Tables

**Table 1:** Clinical manifestations of osteogenesis imperfecta in the study group of children.

Parameter	Patient Number (%)
Low stature	25 (53.2)
Long bones fractures	47 (100)
Vertebrae fractures	31 (66.0)
Skeletal system deformities	32 (68.0)
Osteopenia	16 (34.0)
Osteoporosis	25 (53.2)
Dentinogenesis imperfecta	10 (21.3)
Blue sclerae	41 (87.2)
Hearing disorders	5 (10.6)
Other disorders affecting other systems and organs	10 (21.3)
Orthopedic treatment—Intramedullary anastomosis with Rush rods, Kirchner wires, and Fassier–Duval rods	28 (59.6)

**Table 2:** Anthropometric characteristics in children with osteogenesis imperfecta.

	n	M	SD	Me	Q_1_	Q_3_	Min.	Max.
Weight [kg]	47	25.18	14.72	18.00	15.15	33.00	9.70	73.00
Height/length [cm]	47	115.09	24.52	113.00	96.80	128.50	53.00	175.00
BMI	47	17.90	5.18	15.95	14.63	20.04	11.89	37.35

n—number of patients, M—arithmetic mean, SD—standard deviation, Me—median, Q1—lower quartile, Q3—upper quartile, Min.—lowest value obtained, Max.—highest value obtained.

**Table 3:** Results of genetic testing in a group of children with osteogenesis imperfecta.

Genetic Test Result	Number of Patients (n)	% Group *
*COL1A1*	27	65.9
*COL1A2*	10	24.4
*IFITM5*	3	7.3
*SERPINF1*	1	2.4

*—% of the group calculated based on 41 patients (no data available for the genetic test results for 6 patients).

**Table 4:** Relationship between FGF23 concentration and anthropometric parameters, clinical manifestations, and bone mineral density in the studied group of children with osteogenesis imperfecta.

Parameter	Correlation with FGF23	
	r	*p*
Age	**−0.32**	**0.030**
Number of fractures	−0.05	0.749
Weight	−0.16	0.276
Height	−0.12	0.429
BMI	**−0.34**	**0.020**
Z-score Spine	−0.01	0.945
Z-score TBLH	0.05	0.737
Number of cycles	−0.08	0.580

r—Spearman’s correlation coefficient; statistically significant results are in bold.

**Table 5:** Relationship between FGF23 concentration and the type of osteogenesis imperfecta in the study group of children with osteogenesis imperfecta.

OI Type	n	FGF23 [pg/mL]		Test Statistic	*p*
		Median	Q_1_–Q_3_		
I	24	596,00	258,00–1,269,00	χ^2^ = 5.22 df = 3	0.156
III	16	349,00	214,00–790,00		
IV	3	167,00	152,00–255,00		
V	3	179,00	166,00–444,00		

One patient with type 6 OI was excluded from the analysis. n—number of patients in the subgroup, Q_1_—lower quartile, Q_3_—upper quartile, χ^2^—Kruskal–Wallis test statistic value, df—number of degrees of freedom.

## Data Availability

Data available on request due to restrictions (e.g., privacy, legal or ethical reasons) The data presented in this study are available on request from the corresponding author due to patient privacy and ethical considerations.
